# Systematic proximal mapping of the classical RAD51 paralogs unravel functionally and clinically relevant interactors for genome stability

**DOI:** 10.1371/journal.pgen.1010495

**Published:** 2022-11-14

**Authors:** Estelle Simo Cheyou, Jacopo Boni, Jonathan Boulais, Edgar Pinedo-Carpio, Abba Malina, Dana Sherill-Rofe, Vincent M. Luo, Christophe Goncalves, Halil Bagci, Alexandra Maters, Raquel Cuella-Martin, Yuval Tabach, Sonia del Rincon, Jean-Francois Côté, Barbara Rivera, Alexandre Orthwein

**Affiliations:** 1 Lady Davis Institute for Medical Research, Segal Cancer Centre, Jewish General Hospital, Montreal, Canada; 2 Gerald Bronfman Department of Oncology, McGill University, Montreal, Canada; 3 Bellvitge Biomedical Research Institute, IDIBELL, Barcelona, Spain; 4 Institut de Recherches Cliniques de Montréal (IRCM), Montreal, Canada; 5 Division of Experimental Medicine, McGill University, Montreal, Canada; 6 Department of Developmental Biology and Cancer Research, Institute for Medical Research-Israel-Canada, Hebrew University of Jerusalem, Jerusalem, Israel; 7 Department of Microbiology and Immunology, McGill University, Montreal, Canada; 8 Department of Anatomy and Cell Biology, McGill University, Montreal, Canada; 9 Department of Human Genetics, McGill University, Montreal, Canada; 10 Département de Biochimie et Médecine Moléculaire, Université de Montréal, Montreal, Canada; 11 Département de Médecine (Programmes de Biologie Moléculaire), Université de Montréal, Montreal, Canada; The Univ. of Texas.at Austin, UNITED STATES

## Abstract

Homologous recombination (HR) plays an essential role in the maintenance of genome stability by promoting the repair of cytotoxic DNA double strand breaks (DSBs). More recently, the HR pathway has emerged as a core component of the response to replication stress, in part by protecting stalled replication forks from nucleolytic degradation. In that regard, the mammalian RAD51 paralogs (RAD51B, RAD51C, RAD51D, XRCC2, and XRCC3) have been involved in both HR-mediated DNA repair and collapsed replication fork resolution. Still, it remains largely obscure how they participate in both processes, thereby maintaining genome stability and preventing cancer development. To gain better insight into their contribution *in cellulo*, we mapped the proximal interactome of the classical RAD51 paralogs using the BioID approach. Aside from identifying the well-established BCDX2 and CX3 sub-complexes, the spliceosome machinery emerged as an integral component of our proximal mapping, suggesting a crosstalk between this pathway and the RAD51 paralogs. Furthermore, we noticed that factors involved RNA metabolic pathways are significantly modulated within the BioID of the classical RAD51 paralogs upon exposure to hydroxyurea (HU), pointing towards a direct contribution of RNA processing during replication stress. Importantly, several members of these pathways have prognostic potential in breast cancer (BC), where their RNA expression correlates with poorer patient outcome. Collectively, this study uncovers novel functionally relevant partners of the different RAD51 paralogs in the maintenance of genome stability that could be used as biomarkers for the prognosis of BC.

## Author summary

DNA double-strand breaks (DSBs) are highly cytotoxic DNA lesions that can compromise genomic instability, thereby driving carcinogenesis. DNA repair by homologous recombination (HR) is critical in faithfully repairing this type of DNA damage and the RAD51 paralogs (RAD51B, RAD51C, RAD51D, XRCC2, and XRCC3) have emerged as critical regulators of this pathway. Still, it remains largely unclear how they promote HR-mediate DNA repair. Here, we mapped their respective proximal interactome using the BioID approach and we identified the spliceosome machinery as an integral component of our proximal mapping. Importantly, several members of the spliceosome machinery have prognostic potential in breast cancer (BC), where their RNA expression correlates with poorer patient outcome.

## Introduction

DNA double-strand breaks (DSBs) are highly cytotoxic DNA lesions that can be caused by both exogenous and endogenous sources, including topological stress during DNA replication. In fact, replication stress-induced chromosome breakages are considered the main cause of spontaneous DSBs [1], thereby compromising genomic stability while driving carcinogenesis. Cells have evolved a plethora of complex networks that detect, signal and ultimately repair these threats, such as the high-fidelity homologous recombination (HR) pathway. Importantly, germline mutations in HR genes, such as *BRCA1*, *PALB2*, *BRCA2*, alongside the RAD51 paralogs *RAD51B*, *RAD51C* and *RAD51D* promote the development of hereditary breast and ovarian cancer (HBOC) [2], highlighting the antitumorigenic role of this pathway. Moreover, cross-talks between HR and DNA replication have been recently established [3,4], in particular the protection and the remodeling of stalled replication forks, pointing toward a more complex contribution of the HR pathway in the maintenance of genome stability.

HR is considered a faithful DNA repair pathway as it relies on the use of a homologous donor sequence as a template for the resolution of DSBs [5]. HR is therefore restricted to the S/G2 phases of the cell cycle. For HR to proceed, an extended tract of single-stranded DNA (ssDNA) with a 3’overhang needs to be generated and coated by the replication protein A (RPA), in a process called DNA end resection. The PALB2-BRCA2 complex subsequently promotes the replacement of RPA by the recombinase RAD51 [6–8], thereby allowing the formation of a nucleoprotein filament competent for homology search of complementary sequences and D-loop formation. Several additional players have been shown to stimulate RAD51 filament assembly, including the RAD51 paralogs RAD51B, RAD51C, RAD51D, XRCC2 and XRCC3 [9–12]. These structurally related factors do not directly promote homology search and strand exchange; rather, they are thought to stabilize the RAD51 nucleoprotein filament and facilitate the core recombinase complex [13].

The classical RAD51 paralogs can form two functionally distinct heteromeric complexes: the RAD51B-RAD51C-RAD51D-XRCC2 (BCDX2) and the RAD51C-XRCC3 (CX3) complexes [14]. Biochemical characterization of the BCDX2 has shown that it can bind ssDNA and nicks in duplex DNA [14]. Subsequent *in cellulo* characterization pointed towards a role of the BCDX2 in strand invasion and homology search [11,13]. On the other hand, the CX3 complex has been thought to contribute downstream of RAD51 recruitment in the HR pathway [15]. Importantly, these two sub-complexes also display differential functional roles during the response to replication stress [16]. While the BCDX2 complex restrains fork progression and promotes fork reversal, the CX3 complex mediates efficient reversed fork restart. Still, little remains known about the molecular insight regulating the classical RAD51 paralogs during both DNA repair by HR and the response to replication stress.

To better understand the contribution of the classical RAD51 paralogs *in cellulo*, we mapped their proximal interactome under physiological and replication stress conditions using the BioID approach [17]. Combining our mass spectrometry-based approach with publicly available CRISPR chemogenic and in-house essential screens enabled us to validate a subset of biological complexes associated to classical RAD51 paralogs, including the spliceosome and RNA metabolic processes. Importantly, several members of these pathways have prognostic potential in BC. Altogether, our study provides novel molecular insights into the classical RAD51 paralogs during the maintenance of genome stability.

## Experimental procedures

### Cell culture and treatment

HEK293 Flp-In T-Rex (kind gift from Dr Anne-Claude Gingras, Lunenfeld-Tanenbaum Research Institute) and RPE1-hTERT cells were cultured in Dulbecco’s Modified Eagle medium (DMEM; Wisent) and were supplemented with 10% fetal bovine serum and 1% Penicillin-Streptavidin. All cell lines were tested for mycoplasma contamination and STR DNA authenticated. The following drug was used in this study: hydroxyurea (HU, 4mM; Sigma).

### Plasmids

Human RAD51 paralogs cDNAs were obtained from Mission library clones (McGill University, Sigma) and corresponding BioID constructs were generated via gateway cloning into the pDEST-BirA*-Flag-pcDNA5-FRT-TO (kind gift from Dr Anne-Claude Gingras, Lunenfeld-Tanenbaum Research Institute) following the manufacturer protocol (Invitrogen) and were verified by Sanger sequencing.

### Generation of stable inducible cell lines

Stable inducible cell lines used for all BioID-MS experiments were generated in HEK293 Flp-In T-REx cells following the manufacturer protocol (Invitrogen). Briefly, cells were seeded at a density of 3.65 x 10^5^/100mm plate in 10mL growth media. The next day, cells were transfected with 5μg of the indicated BirA*-tagged construct and 1.25μg of the Flp-recombinase expression vector pOG44 using Lipofectamine^TM^ 2000 (Invitrogen) as recommended by the manufacturer. Transfected cells were subsequently transferred into 150mm plates and selected for positive transfection by the addition of 200μg/mL hygromycin and 5μg/mL blasticidin to the growth media. Cells were maintained under selection media for 2–3 weeks until clear colonies appeared. Upon colony formation, cells were pooled and screened for stable expression by western blotting after tetracycline induction and biotin labelling.

### Stable cell line validation by western blot

Expression of the indicated BirA*-tagged construct and biotin labelling were performed as described previously [18]. Stable cell lines were lyzed in in RIPA buffer (50mM Tris-HCl pH 7.5, 150mM NaCl, 1% (v/v) NP-40, 1mM EDTA, 1mM EGTA, 0.1% SDS and 0.5% sodium deoxcycholate, 1mM PMSF, 1 mM DTT) and proteins were separated by SDS-PAGE and transferred onto nitrocellulose membranes. The membranes were blocked in TBS containing 5 mg/mL non-fat milk and 1% Tween 20 for 1 hour at room temperature. Blots were probed for Flag (Sigma), α-tubulin (NEB), and streptavidin conjugated to HRP (GE Healthcare).

### Sulforhodamine B (SRB) Assay

HEK293 Flp-In T-REx cells were seeded in 96-well plates at a density of 1000/cells per well. Twenty-four hours later, cisplatin was added in a two-fold serial dilution from 50 to 0.097 μM. Survival was assessed four days after treatment using the sulforhodamine B (SRB) colorimetric assay. Briefly, after drug treatment cells were fixed by adding 100 μL of 10% trichloroacetic acid (TCA, Bioshop Canada) and incubated at 4°C for 1 hr with gentle agitation. Cells were washed four times and plates were left air-drying overnight at room temperature. The next day, cells were stained with 100 μL of 0.057% SRB (Sigma Aldrich) and incubated at room temperature for 30 minutes. Plates were then rinsed four times using 1% acetic acid and were left air-drying overnight. Protein content was solubilized by adding 200 μL of 10 mM Tris base solution (pH 10.5) for 30 minutes at room temperature. Measurement of optical density (OD) at 510 nm was conducted using a FLUOstar Optima microplate reader. Background correction was conducted using the measurement of control wells with media. Treatments were performed in triplicate, averaged, and normalized to untreated control. IC50 concentrations were obtained using the slope’s equation for log(concentration of drug) vs normalized OD.

### BioID sample preparation for mass spectrometry

2 × 150mm plates of cells were used per replicate. Induction of fusion protein expression and biotin labelling were performed as described previously [18]. Cell pellets were lysed in RIPA buffer and sonicated on ice at an amplitude of 30% and a rate of 3 × 10 s. bursts with 3 s. rest in between. After sonication, 250 units of benzonase (EMD) were added to each sample, and cell lysates were vigorously vortexed and centrifuged for 30 min at 12000 rpm at 4°C. In the meantime, 30μL of packed streptavidin-Sepharose beads (GE Healthcare) were washed in RIPA buffer. After centrifugation, each lysate supernatant was added to pre-washed beads and biotinylated proteins were captured by affinity at 4°C on a rotator for 3 hr. Subsequently, the beads were pelleted at 2000 rpm for 1 min and washed twice with 1mL RIPA and three times with 50mM ammonium bicarbonate (ABC, pH 8.0). After the last wash, beads were resuspended in 100μL of ABC and on-bead digestion was achieved by adding 10μg of mass spectrometry-grade trypsin (Sigma) to the suspension for overnight incubation at 37°C on a rotating disc. The next day an additional 10μg of trypsin were added to each sample followed by an additional 3hr incubation at 37°C with rotation. Supernatants enriched with peptides were collected by centrifugation and pooled with supernatants from two subsequent rinses with 100μL of HPLC-grade H_2_O. 50% formic acid was added to the pooled samples to a final concentration of 2% (v/v) to end digestion before centrifugation at 12000 rpm for 10 min. Supernatants containing the peptides were transferred into new tubes and dried in a centrifugal evaporator for 3 hr at high rate. Peptides were resuspended in 15μL of 5% (v/v) formic acid and kept at -80°C until further analysed by mass spectrometric analysis.

### Mass spectrometry data analysis

Samples were injected into an Orbitrap Fusion (Thermo Fisher), and raw files were analyzed with the search engines Mascot using the human RefSeq database (version 20170518) supplemented with “common contaminants” from the Max Planck Institute (http://maxquant.org/downloads.htm), the Global Proteome Machine (GPM; http://www.thegpm.org/crap/index.html) and decoy sequences. The search parameters were set with trypsin specificity (two missed cleavage sites allowed), variable modifications involved Oxidation (M) and Deamidation (NQ). The mass tolerances for precursor and fragment ions were set to 15 ppm and 0.6 Da, respectively, and peptide charges of +2, +3, +4 were considered. Search results were individually processed by PeptideProphet [19], and peptides were assembled into proteins using parsimony rules first described in ProteinProphet [20] using the Trans-Proteomic Pipeline (TPP). TPP settings were the following: -p 0.05 -x20 -PPM–d “DECOY”, iprophet options: pPRIME and PeptideProphet: pP. Only proteins having at least one unique peptide and an iProphet probability ≥ 0.9 were considered. Prior to the bioinformatics analyses, we discarded proteins considered as classical BioID contaminants, such as carboxylases, lysozyme, keratins, ribosomal subunits, AHNAK, PRKDC, TOP1, HLCS, FLNB, PRKAA1, PRKAA2.

From the preys identified by our five baits, an upset plot was created with the upsetR package [21] in R. Using the ProHits-Viz online tool (prohits-viz.org), we generated a dot plot representing the relative AvgSpec of identified subunits among the BCDX2, CX3 and the PALB2-RAD51-RAD51C-BRCA2 complexes. Heatmaps were generated in R (r-project.org) with the ComplexHeatmap package [22] by performing hierarchical clustering on drugs NormZ-scores derived from published CRISPR screens [23,24] from preys identified by our BioID assays. Similarly, we created heatmaps out of our BioID results using the log2-transformed SAF (Spectral Abundance Factor) metric, a normalization method calculated by dividing average spectral counts of preys by their respective protein length in amino acids. Preys unidentified by our RAD51 baits were imputed with an average spectral count of zero. We next calculated log_2_ fold changes of average spectral counts of preys identified in HU-treated cells over untreated cells by using the following formula: log_2_(HU+1/Untreated+1). Overrepresentation analyses of GO terms, KEGG pathways and CORUM complexes were executed with the R package gprofiler2 [25] by excluding electronic annotations and statistically correcting p-values with the false discovery rate (fdr) correction method. We selected statistically significant terms and presented the results in dotplots with the ggplot2 R package. Graphical representations of protein–protein interaction networks were generated by importing our BioID results in Cytoscape (v.3.9.1) (cytoscape.org). Finally, we performed a network augmentation by extracting prey–prey interactions from the human BioGRID database (v.4.4.203) [26], and from Cytoscape’s PSICQUIC Web Service client (November 2021 release) through the IntAct, iRefIndex and UniProt databases.

### CRISPR genome-wide essential screen

CRISPR-based genome-wide screen was completed as detailed in [27]. Briefly, 270 x 10^6^ RPE-hTERT cells stably expressing the nuclease Cas9, were transduced with TKOv1 concentrated library virus at MOI = 0.2, ensuring a coverage of at least 600-fold for each individual sgRNA represented in the cell population. Two days later, puromycin (15μg/ml) was added to the media to allow for the emergence of resistant cells. At initial and end time points, cell pellets were collected and frozen prior to genomic DNA extraction. Cell pellets were resuspended in 6 mL DNA lysis buffer (10mM Tris-Cl, 10mM EDTA, 0.5% SDS, pH 8.0) with 100μg/mL RNaseA, followed by incubation at 37°C for 60 min. Proteinase K was subsequently added and lysates were further incubated at 55°C for two hours. Samples were then briefly homogenized before being transferred into pre-spun 15 mL MaXtract tubes (Qiagen) mixed with an equal volume of neutral phenol:chlorophorm:isoamyl alcohol (25:24:1) solution, shook and centrifuged at 1,500g for 5 min at room temperature. The aqueous phase was extracted and precipitated with two volumes of ethanol and 0.2M NaCl. Air-dried pellets were resuspended in water and quantitated via UV absorbance spectrometry. Preparation for next-generation sequencing (NGS) was completed as described previously [27]. Prior to analysis, FastQ NGS read files were processed using FastQC software to assess uniformity and quality. Reads were trimmed of NGS adapter sequences and reads were aligned to the sgRNA library index file using Bowtie2 to assign a matching gene-specific sgRNA. BAM files were generated using samtools and total read count tables were subsequently generated using MAGeCK count command. DrugZ algorithm was used to identify gene knockouts which were depleted or enriched from day 14 (D14) populations in comparison to day 0 (D0) [28].

### Phylogenetic profiling of selected preys

The CladeOScope [29] was used to predict functional interactions between the RAD51 paralogs and selected preys identified by our proximal mapping. *HIRA* and *CHERP* co-evolved with known DNA repair genes in the Ascomycota phylum from the Fungi kingdom that englobes 323 different species. The co-evolved DNA repair genes were identified through enrichment analysis of the top 50 co-evolved genes in the Ascomycota clade phylum using EnrichR and GeneAnalytics.

### Kaplan Meier and differential gene expression analyses

Kaplan-Meier plotter (kmplot.com/analysis) was used to correlate the gene expression (RNA-seq, gene chip) of proximal interactors for the different RAD51 paralogs with breast cancer patient outcome. Only genes showing a significant correlation (p-value<0.05) were represented.

### Experimental design

Two biological replicates were completed for each bait and each experimental condition (untreated and HU). All BioID samples were run on the Orbitrap Fusion (Thermo Fisher) at the same to limit batch effect and allow side-by-side comparison of the different baits.

## Results

### Proximal mapping of the classical RAD51 paralogs

**Fig 1:**
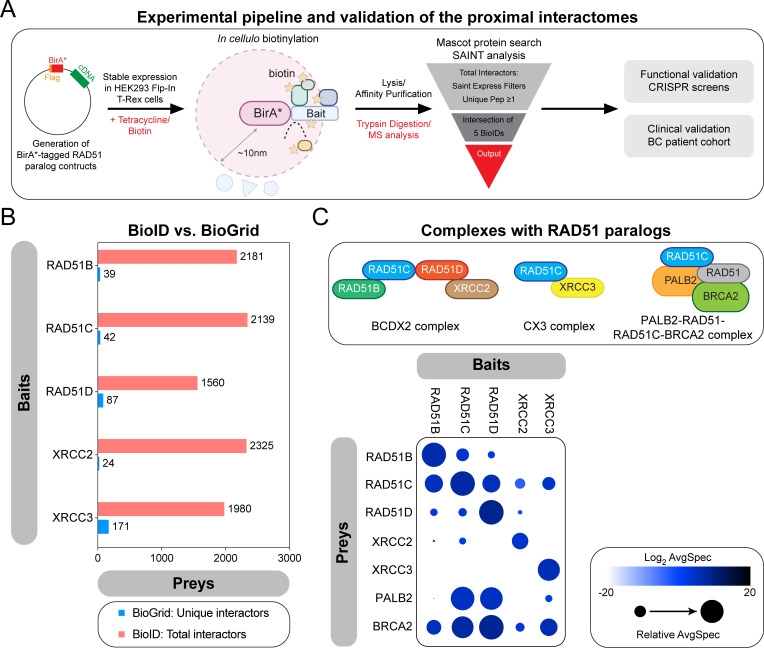
Proximal mapping of the RAD51 paralogs identifies known sub-complexes. (A) Schematic representing the experimental pipeline developed to map the proximal interactomes of the classical RAD51 paralogs and their subsequent functional and clinical validations. (B) Representation of the number of preys identified in each BioID in comparison to curated unique interactors annotated in the BioGRID database. (C) Top: schematic representing the well-established sub-complexes formed by the classical RAD51 paralogs. Bottom: selected BioID results, shown as dot plots. The spectral counts for each indicated prey protein are shown as AvgSpec. The circle size represents the relative abundance of preys over baits.

To gain insight into the classical RAD51 paralogs *in cellulo*, we used the BioID labelling technique, which allows the monitoring of proximal/transient interactions [30]. Briefly, the five classical RAD51 paralogs were fused at their N-terminus to an abortive mutant of an *E*.*coli* biotin-conjugating enzyme (BirA*) (Figs 1A and S1A), and stably expressed in the human embryonic kidney 293 (HEK293) cell line using the Flp-In/T-REx system. These fusion proteins are capable of biotinylating factors that come in proximity or directly interact with the classical RAD51 paralogs (S1B Fig). We also tested the functionality of the tagged RAD51 paralogs using the SRB assay (S1C Fig). Labelled proteins were subsequently purified by streptavidin affinity and identified by mass spectrometry. This labelling was performed in absence of any genotoxic stress to map the proximal interactome of the classical RAD51 paralogs at steady state. For each bait, we identified more than 1500 unique proximal interactors (Fig 1B, S1 Table), a ~10- to a 100-fold increase compared to previous reports compiled by BioGrid [26], highlighting the power of the BioID approach.

To ensure that our BirA*-fused constructs mapped physiological interactions, we first focused our attention on well-described molecular complexes formed by the five classical RAD51 paralogs: the BCDX2, the CX3 and the RAD51C-PALB2 complexes (Fig 1C). Proximal mapping of each bait identified the different components of both BCDX2 and CX3 complexes: for instance, the proximal interactome of RAD51B mapped all other classical RAD51 paralogs, except XRCC3 (Fig 1C), while XRCC3 BioID only contains RAD51C. In line with these findings, we noted that PALB2 is part of the RAD51C BioID, thereby validating our approach. As anticipated, BRCA2 was part of the proximal interactome of all classical RAD51 paralogs (Fig 1C), confirming their epistatic relationship [31]. Interestingly, we noticed that PALB2 is part of the proximal interactomes of several classical RAD51 paralogs (Fig 1C), in particular RAD51D, suggestive of more complex relationship between PALB2 and the different paralogs than previously identified [32]. Altogether, these data highlight the power of the BioID approach in mapping the proximal interactome of the classical RAD51 paralogs at steady state.

### Differential functional proximal interactomes between the BCDX2 and CX3 complexes

**Fig 2:**
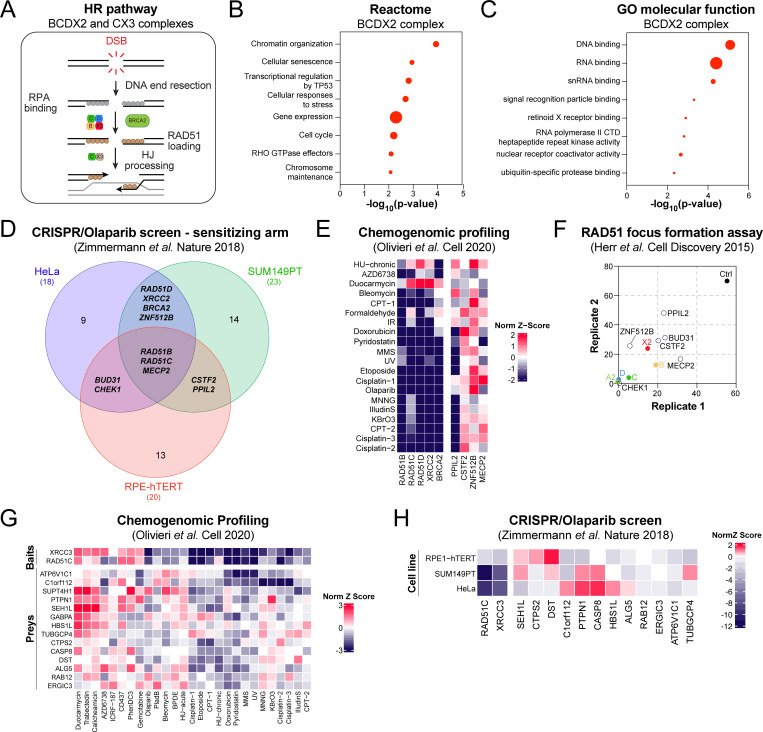
Proximal mapping of the BCDX2 and CX3 complexes. (A) Schematic representing the different steps of the HR pathway and the sequential involvement of the BCDX2 and CX3 complexes during this process. (B) Pathway enrichment analysis of the preys that are exclusive to the BCDX2 complex using the Reactome database. Data are represented as the -log_10_ of the p-value (log_10_(p-value)) calculated for each indicated pathway by Reactome. Each dot is proportional to the number of preys that belong to the indicated pathway. (C) GO molecular function analysis of the preys that are exclusive to the BCDX2 complex. Data are represented as the -log_10_ of the p-value (log_10_(p-value)) calculated for each indicated pathway by GO algorithm. Each dot is proportional to the number of preys that belong to the indicated pathway. (D) Venn diagram representing the overlap of the CRISPR screens published by [24] where sensitivity to the PARPi olaparib was tested in three different cell lines. Only the genes whose inactivation by CRISPR provided a significant sensitization to olaparib (NormZ-score<1) are plotted. (E) Heatmap clustering representing the NormZ-scores of our selected preys alongside the classical RAD51 paralogs in a series of CRISPR screens published by [23]. (F) Representation of the percentage of S/G2 U2OS cells transfected with the indicated siRNA and displaying more than 9 IR-induced RAD51 foci as published in [44]. Each replicate is plotted on the x- and y-axis. (G) Heatmap clustering representing the NormZ-scores of our selected preys alongside RAD51C and XRCC3 in a series of CRISPR screens published by [23]. (H) Heatmap clustering representing the NormZ-scores of our selected preys alongside RAD51C and XRCC3 in a series of CRISPR screens published by [24] where sensitivity to the PARPi olaparib was tested in three different cell lines.

The heteromeric BCDX2 and CX3 complexes have been shown to play sequential roles in the HR pathway (Fig 2A) [13,15]. However, it remains largely unclear how they mediate their function during this process. Thus, we intersected the BioID of the 5 classical RAD51 paralogs and focused our attention on 97 common preys of the BCDX2 complex (S2A Fig, S2 Table). Interestingly, this analysis identified the serine/threonine-protein kinase CHEK1, alongside several of its previously established functional partners (e.g. MHL1, POLA1, PLK1) [33–35] as proximal interactors of the BCDX2 complex (S2B Fig), likely reflective of the well described role of ATR-CHEK1 signaling in the regulation of RAD51 paralogs [36,37]. Reactome analysis of these 97 preys identified chromatin organization (p-value = 1.16x10^-4^), cellular senescence (p-value = 1.13x10^-3^), and transcriptional regulation by TP53 (p-value = 1.53x10^-3^) as top enriched pathways (Fig 2B, S3 Table), while gene ontology (GO) molecular function analysis revealed a significant enrichment in DNA and RNA binding proteins (Fig 2C, S3 Table). How these proximal interactors of the BCDX2 complex may participate in the maintenance of genome stability remain elusive.

To evaluate their relevance to the HR pathway, we interrogated 3 publicly available omics datasets where the relationship of a given human gene to this pathway has been systematically assessed by either CRISPR or RNA interference (RNAi) technology [23,24,38]. First, we took advantage of a dataset where CRISPR-based genome-wide dropout screens were completed in either neoplastic (HeLa and SUM149PT) or non-transformed (RPE1-hTERT) cell lines using the PARP inhibitor (PARPi) olaparib as a surrogate for HR deficiency [24]. CRISPR-mediated inactivation of the BCDX2 complex or *BRCA2* sensitized to olaparib in at least two cell lines (NormZ-score<-1; Fig 2D, S3 Table). Similarly, 63 out of the 97 proximal interactors of the BCDX2 complex significantly impacted the response to olaparib in at least one cellular background (Figs 2D and S2C and S3 Table). Of note, 22 out of 97 preys did not score in this dataset, thereby providing a validation rate of 84%.

Our subsequent analysis focused on the 6 preys (ZNF512B, MECP2, BUD31, CHEK1, CSTF2, PPIL2) that provided sensitivity to olaparib in, at least, two cell lines (Fig 2D, S3 Table), and evaluated their chemogenomics profile in a series of CRISPR-based screens completed in RPE1-hTERT cells against different genotoxic agents [23]. We limited our analysis to a total of 20 CRISPR screens where the components of the BCDX2 complex were preferentially enriched (S3 Table). Strikingly, we noticed that inactivation of the RING-type E3 ubiquitin ligase PPIL2 correlated with an increased sensitivity to several genotoxic agents that rely on the HR pathway for their processing and repair (Fig 2E, S3 Table), including cisplatin, camptothecin (CPT), etoposide, bleomycin, and the alkylating agent methylnitrosoguanidine (MNNG). PPIL2 has been previously shown to interact with the zinc finger protein ZNF830 [39,40], a modulator of the HR pathway [41], and our data suggest that this E3 ubiquitin ligase may also participate in the regulation of the BCDX2 complex during DNA repair. To test this hypothesis, we interrogated the relevance of PPIL2, alongside ZNF512B, MECP2, BUD31, CHEK1, and CSTF2, in a publicly available dataset that tested the genetic dependencies linked to the formation of ionizing radiation (IR)-induced RAD51 foci [42]. As control, we used the components of the BCDX2 complex and BRCA2, which are known to impair RAD51 focus formation (Fig 2D, S3 Table). Interestingly, depletion of our 6 predictions by RNA interference significantly impaired the formation of IR-induced RAD51 foci in U2OS cells (Fig 2F, S3 Table).

We extended our targeted analysis to the CX3 complex by focusing on preys that were exclusively identified in the BioID of both RAD51C and XRCC3, thereby identifying 46 proximal interactors of the CX3 complex (S2A Fig, S2 Table). Chemogenomic profiling of these preys identified a subset of 14 factors, whose inactivation by CRISPR resulted in a response to a series of DNA damaging agents that shares similarities to XRCC3 or RAD51C depletion (Figs 2G and S2D and S4 Table). This approach delineated C1orf112 as an exclusive partner of the CX3 complex, in line with a recent report linking C1orf112 to DNA repair pathways [43]. Importantly, CRISPR-mediated inactivation of C1orf112 sensitized to olaparib in different cellular backgrounds (e.g., RPE1-hTERT and SUM149PT; Fig 2H, S4 Table). Altogether, our proximal mapping identified novel functional effectors of both BCDX2 and CX3 complexes, in keeping with their differential contribution to the HR pathway (S2E Fig).

### Proximal mapping of the RAD51 paralogs identified the spliceosome as relevant for HR

**Fig 3:**
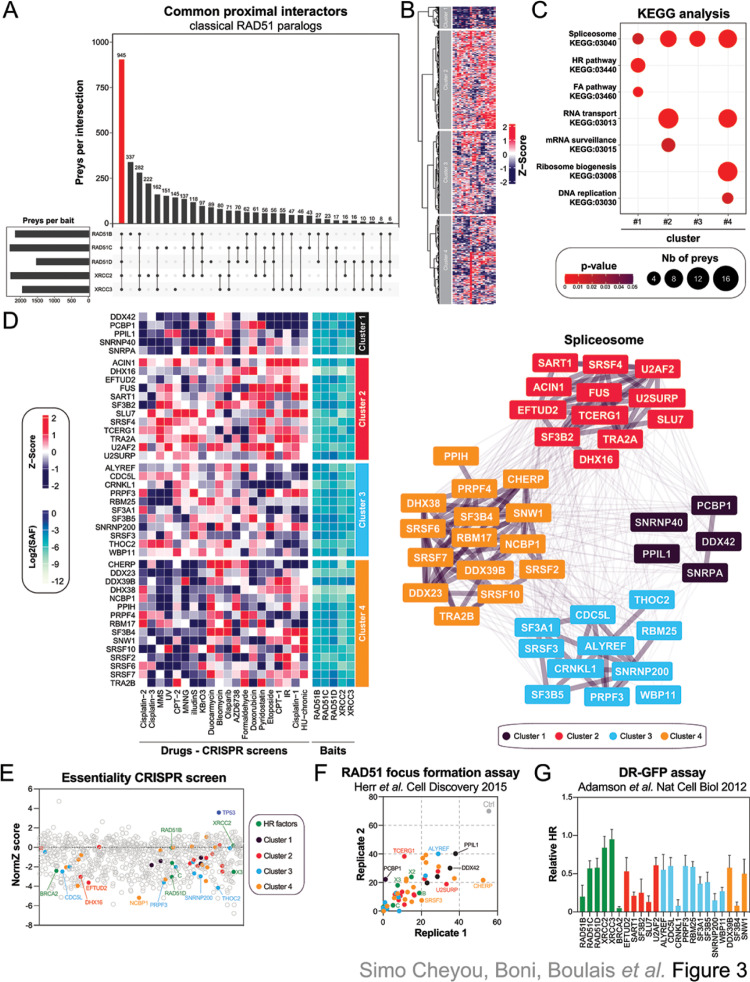
Identification of the spliceosome as a regulator of the classical RAD51 paralogs. (A) Representation of the overlap between the BioID of the different RAD51 paralogs. (B) Heatmap clustering representing the NormZ-scores of the common preys of the RAD51 paralogs in a series of CRISPR screens published by [23] where different genotoxic drugs were tested in RPE1-hTERT cells. (C) Pathway enrichment analysis of the preys that are common to all classical RAD51 paralogs using the KEGG database. Pathways are represented based on their p-value calculated by KEGG algorithm for the 4 different clusters. Each dot is proportional to the number of preys that belong to the indicated pathway. (D) Representation of the different spliceosome factors identified in each cluster. (E) Representation of the CRISPR-based essential screen performed in RPE1-hTERT cells. Each common prey of the classical RAD51 paralogs is represented by its respective NormZ-score. (F) Representation of the percentage of S/G2 U2OS cells transfected with the indicated siRNA and displaying more than 9 IR-induced RAD51 foci as published in [44]. Each replicate is plotted on the x- and y-axis. (G) Representation of the relative HR monitored in U2OS DR-GFP cells transfected with the indicated siRNA as published in [38].

To further understand the contribution of the classical RAD51 paralogs *in cellulo*, we intersected their respective BioID, thereby identifying 945 common proximal interactors (Fig 3A, S5 Table). Clustering of their respective CRISPR chemogenomic profile identified 4 different sub-groups (Fig 3B). As expected, the classical RAD51 paralogs, alongside well-established FA/HR factors, such as BRCA2, FANCI, RAD50, BLM and POGZ, segregated in the same cluster (cluster 1, Figs 3B and S3A). In line with these findings, pathway enrichment analyses identified DNA repair pathways and cell cycle pathways as significantly enriched in cluster 1 (Figs 3C and S3B and S6 Table), thereby validating our approach.

We extended our KEGG analysis to the different chemogenomic clusters to map the molecular networks associated with the classical RAD51 paralogs (S7 Table). The spliceosome machine (KEGG:03040) emerged as a common pathway of the chemogenomic sub-groups (Fig 3C, S6 Table). Indeed, we noted that several splicing factors are proximal interactors of the classical RAD51 paralogs at steady state and their targeting by CRISPR technology significantly modulates the response to genotoxic drugs (Fig 3D), in line with phylogenetic co-evolution profiling data [44]. RAD51 paralogs are known to be essential in non-transformed human cells [13]. Thus, we wondered whether our preys may provide a similar phenotype in RPE1-hTERT cells and performed a systematic mapping of “essential” genes by CRISPR technology (S3C Fig). As expected, targeting of TP53 promotes growth of RPE1-hTERT cells while deletion of RAD51C, RAD51D, XRCC3 or BRCA2 impaired cell survival *in vitro* (Fig 3E), validating our approach. In total, CRISPR-mediated deletion of 305 common preys decreased RPE1-hTERT proliferation *in vitro* (Norm Z-score <-1.5; Fig 3E, S8 Table), including 28 out of the 43 splicing factors that we previously identified by chemogenomic profiling. We independently validated our observations using previous omics datasets that directly assayed DNA repair by HR, using both the formation of IR-induced RAD51 foci and the DR-GFP assay as read-outs (Figs 3F, 3G and S3D) [38,42]. Strikingly, depletion of all 43 splicing factors by RNA interference significantly impaired the formation of RAD51 foci in U2OS cells (Fig 3F), and several of them drastically decreased HR potential in the DR-GFP assay (Figs 3G and S3E). Altogether, our data pointed towards a complex regulation of the classical RAD51 paralogs during the maintenance of genome stability, with a key contribution of the spliceosome in this process.

### RNA metabolic processes collaborate with the RAD51 paralogs during replication stress

The classical RAD51 paralogs have been recently implicated in the response to replication stress [16]. This prompted us to map their proximal interactome under replication stress conditions by exposing our stable HEK293 cell lines to hydroxyurea (HU), which depletes deoxyribonucleotide pools and immediately stalls replication forks [45]. Most preys that we identified for each classical RAD51 paralog under normal conditions were also present upon treatment with HU (S4A Fig, S9 Table), which may reflect the lengthy labeling pulse required for our BioID approach [46].

**Fig 4:**
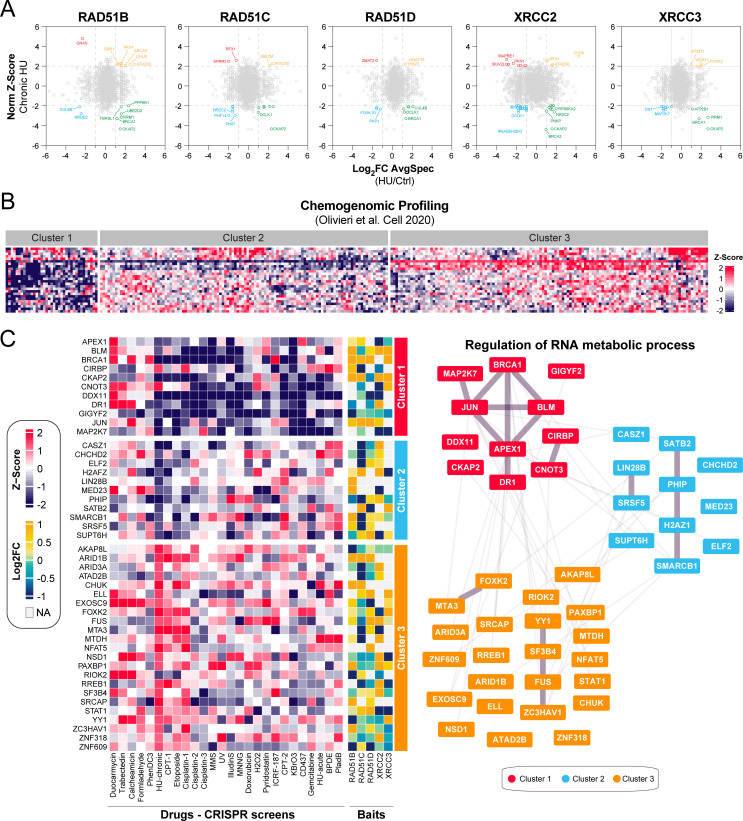
RNA metabolic process collaborates with the RAD51 paralogs upon replication stress. (A) Differential BioID analysis of the classical RAD51 paralogs upon HU exposure intersected with a CRISPR-based genome screen where sensitivity to a chronic HU treatment was tested [23]. Data are represented as the log_2_FoldChange (log_2_FC) of the average peptide count for a given prey between HU and untreated conditions on the x-axis and the NormZ-score for the HU-chronic CRISPR screen published in [23]. Significant differentially modulated proximal interactors are considered for those with log_2_FC<1 or >1 and NormZ-score<-2 or >2. (B) Heatmap clustering representing the NormZ-scores of the preys significantly modulated identified in (A) and monitored in a series of CRISPR screens published by [23] where drugs inducing replication stress were tested in RPE1-hTERT cells. (C) Representation of the different factors involved in RNA metabolic process and identified in each cluster.

To better appreciate dynamics in the proximal interactome of the different RAD51 paralogs under replicative stress conditions, we monitored stress-induced changes in the average spectral count of a given prey and intersected them with previous CRISPR-mediated genome-wide screen where each gene was evaluated for its contribution to a chronic HU treatment (Fig 4A). In total, we identified 323 preys that were differentially present in at least one HU-BioID and that scored significantly in HU CRISPR-based genome-wide screen [23]. As expected, several factors that have been previously involved in replication stress, such as BRCA1, RAD18, PIAS4, and BLM, alongside the ribonucleotide reductase RRM1, were differentially present in the vicinity of multiple RAD51 paralogs upon HU treatment (Fig 4A, S10 Table), validating our approach. Interestingly, we noticed that the doublecortin-like kinase DCLK1 (also known as DCAMKL1) is enriched in our BioIDs under replication stress. Various studies have demonstrated the importance of DCLK1 during the DNA damage response [47,48], and our data suggest that DCLK1 may play a role in the regulation of the classical RAD51 paralogs during the response to replication stress.

To better grasp how these factors may participate in the response to replication stress, we performed a similar chemogenomic analysis as previously described, but with a focus on drugs known to cause replication stress. Profiling of our preys of interest delineated three distinct clusters (Fig 4B), and pathway enrichment analysis identified DNA repair (GO:0006281, p = 2.84x10^-9^), and cell cycle (GO:0007049, p = 6.31x10^-8^), as being significantly enriched in cluster 1 (S4B and S4C Figs, S11 and S12 Tables). In line with these findings, we noticed that BRCA1, REV3L, RAD18 and RNASEH2B clustered in this sub-group (S4B Fig).

We hypothesized that cluster 1 may contain novel DNA repair factors relevant for the response to replication stress. To test this hypothesis, we took advantage of the CladeOScope, a clade-wise phylogenetic profiling tool that can predict gene function [29]. Interestingly, we noticed that the histone chaperone HIRA co-evolved with several established DNA repair factors in the Ascomycota phylum (S4D Fig), such as MSH6, RECQL4, RECQL5, and ERCC4. Similarly, the alternative splicing regulator CHERP co-evolved with well described HR factors in the same phylum (e.g., MUS81, EME1, and HELQ), suggestive of a potential contribution of both factors in the maintenance of genome stability under replication stress. In-depth analysis of this cluster 1 identified two RNA-linked factors that have been shown to alter IR-induced RAD51 focus formation (S4E Fig, S13 Table): a component of the mRNA decapping complex, EDC3, and the RNA binding protein CIRBP. More generally, GO analysis of cluster 1 revealed a significant enrichment in factors involved in the positive regulation of RNA metabolic process (GO:0051254, p-value = 6.258x10^-4^) (S4B and S4C Figs, S11 Table). In fact, a total of 45 proximal interactors of the RAD51 paralogs participate in the positive regulation of RNA metabolic process (**Fig 4C**), suggestive of an important role for this pathway during replication stress.

### Proximal interactors of the RAD51 paralogs may have prognostic potential in BC

**Fig 5:**
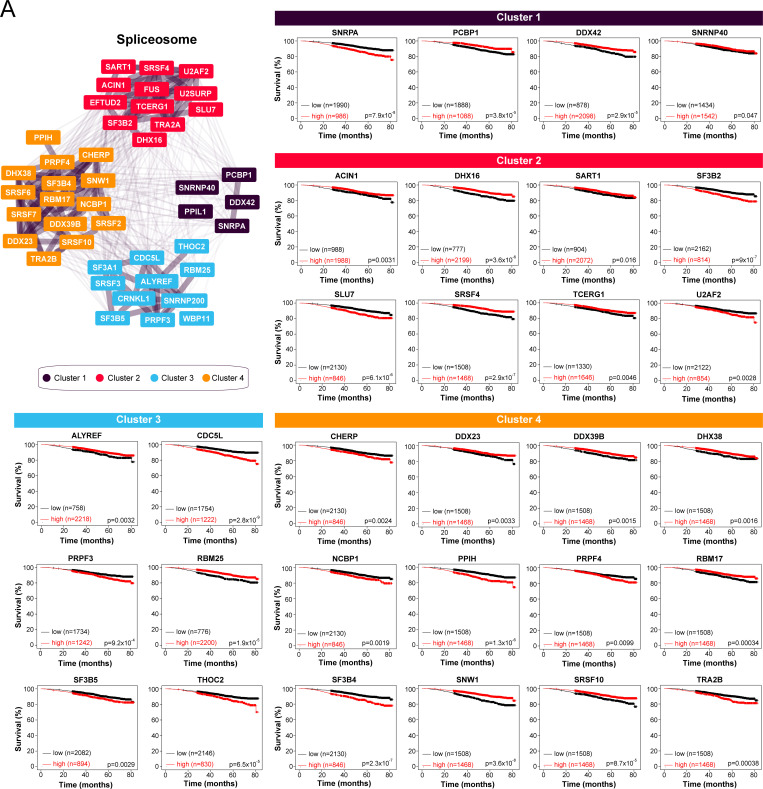
Several splicing factors that are proximal to the RAD51 paralogs have prognostic potential in BC. (A) Overall survival analysis based on the RNA levels of the indicated splicing factors in a cohort of BC patients using KMplot (n = 2976 patients). Only the preys linked to the spliceosome that have a significant impact on BC overall survival (p<0.05) are indicated.

To determine whether our approach can identify clinically relevant proximal interactors of the classical RAD51 paralogs, we focused our attention on the spliceosome and interrogated their RNA expression by RNA sequencing in a publicly available cohort of BC patients (n = 2976) [49]. Interestingly, the expression of 34 out of the 43 proximal interactors linked to the spliceosome correlated with a significant impact on the overall survival of BC patients (Fig 5A, S14 Table), including SNRNP40 (cluster 1), SF3B2 (cluster 2), SF3B5 (cluster 3), and SNW1 (cluster 4). We extended our analysis to another publicly available BC cohort where gene expression was analysed by microchip and linked to relapse-free survival (RFS; n = 4934) and overall survival (OS; n = 1880) [49]. There, the RNA expression of 32 splicing factors correlated with a significant impact on the RFS of BC patients (S14 Table), while 22 of them influenced the OS of BC patients.

We performed a similar analysis with members of the positive regulation of RNA metabolic process, which delineated 31 (out of 43) potential prognostic factors in the cohort of BC patients analyzed by RNA-seq (S5A Fig, S14 Table), including CNOT1 (cluster 1), ELF2 (cluster 2), and PAXBP1 (cluster 3). Subsequent analysis in an independent BC cohort identified 35 members of the positive regulation of RNA metabolic process, whose RNA expression had a significant impact on the RFS of BC patients, while 25 of them influenced their OS (S14 Table). Altogether, these data suggest that the spliceosome and RNA metabolism processes may play an important role for the pathobiology of BC.

## Discussion

The five classical RAD51 paralogs were first identified more than three decades ago by both DNA sequence alignment and functional characterization in hamster cells [9,10,50]. Until recently, it remained largely unclear how these paralogs participated in the maintenance of genome stability, in particular the repair of DSBs by HR. Here, we systematically mapped the proximal interactome of the five classical RAD51 paralogs using the BioID approach, providing new insight into the molecular regulation of these factors at steady state and during replication stress.

Initial biochemical characterization delineated two main subcomplexes formed by the classical RAD51 paralogs: the BCXD2 and the CX3 complexes [14]. Our *in cellulo* proximal mapping confirmed this complex network between the different RAD51 paralogs, alongside their close association with BRCA2. A more recent mass spectrometry-based study identified RAD51C in complex with PALB2, which has been proposed to serve as scaffold for BRCA2 and RAD51 [32]. Our BioID approach detected this stable association but also identified a novel complex composed of both RAD51D and PALB2. Whether this new molecular structure cooperates with BRCA2, alike RAD51C and PALB2, in the repair of DSBs by HR remains to be further investigated. Still, our data suggest that the classical RAD51 paralogs may form previously unidentified molecular networks *in cellulo*, with direct relevance for the maintenance of genome stability. Indeed, our targeted proteomic analysis of the BCDX2 and CX3 complexes identified factors that may promote different steps during DNA repair by HR, in accordance with previous reports that delineated sequential roles for these sub-complexes [13,15]. Several proximal interactors of the BCDX2 complex identified in this study possess DNA and/or RNA binding capacity and have been previously shown to regulate the response to the PARPi olaparib [23,24], and the formation of IR-induced RAD51 foci [42], likely participating directly, or indirectly, the well-established role of the BCDX2 in the loading of RAD51 at processed DSBs [13,15]. On the other hand, mapping of the CX3 proximal interactome identified a poorly characterized open reading frame, C1orf112, as an integral component of the HR pathways as previously suggested [43].

RNA-related processes have emerged as an integral component of the DNA damage response [51,52]. It is well established that the spliceosome machinery controls the transcription of established DNA repair genes [53,54], alongside putative regulators of these factors [44]. However, additional mechanisms of regulation of the DNA damage response have been attributed to the spliceosome machinery and its associated RNA binding proteins, including the signaling of the break, the remodeling of chromatin at DNA damage sites, DNA:RNA hybrids stabilization, RNA-templated DNA repair and liquid-liquid phase separation. In particular, our proximal mapping identified the hnRNP FUS, which have previously shown to play a critical role in the response to ionizing radiation and DNA repair by HR [55–57], suggesting that a potential crosstalk between this splicing factor and the RAD51 paralogs in the maintenance of genome stability. Our analysis further exemplifies the non-canonical contribution of RNA-based processes in the regulation of established DNA repair factors.

Our study suggest that RNA processes are tightly connected to the classical RAD51 paralogs in the maintenance of genome stability. Critically, we show that their gene expression, alike *BRCA1*, influences the prognosis of BC patients, in lines with previous reports identifying splicing dysregulation as a driving force of BC pathobiology (reviewed in [58]). Further studies will be required to define whether these factors predispose to the development of BC, alike several of the classical RAD51 paralogs [2]. Altogether, our data highlight the power of the BioID approach in the identification of novel complexes involved in the maintenance of genome stability, with direct relevance for BC.

## Supporting information

S1 Fig Cell lines validation for BioID. (A) Schematic representing the different classical RAD51 paralogs and their functional domains. (B) HEK293 Flp-In cells stably expressing each BirA*-Flag-tagged RAD51 paralog were tested for expression and biotinylation following induction with tetracycline and incubation with biotin as indicated. After induction, cells were lysed and subjected to immunoblot for streptavidin, flag and α-tubulin. (C) Quantification of HEK293 Flp-In cells stably expressing each BirA*-Flag-tagged RAD51 for their response to cisplatin using the SRB assay. Cisplatin was added at a maximum concentration of 50 μM in a two-fold serial dilution until 0.097 μM. Data are represented as the mean ± SEM (n = 3 independent experiments). HEK293 Flp-In cells transfected with the indicated siRNA were used as positive controls. Data were analyzed using a one-way Welch’s ANOVA test and Dunnett’s multiple comparison tests. *p<0.01
(EPS)

S2 Fig Analysis of the BCDX2 and CX3 proximal interactomes. (A) Venn diagram representing the overlap of the different BioID of the RAD51 paralogs. (B) STRING analysis of CHEK1 and the common preys of the BCDX2 complex. (C) Venn diagram representing the overlap of the CRISPR screens published by [24] where sensitivity to the PARPi olaparib was tested in three different cell lines. Only the genes whose inactivation by CRISPR provided a significant resistance to olaparib (NormZ-score<1) are plotted. (D) Heatmap clustering representing the NormZ-scores of our selected preys alongside RAD51C and XRCC3 in a series of CRISPR screens published by [23]. (E) Schematic placing the novel regulators of the BCDX2 and CX3 complexed in the HR pathway and identified by our BioID approach.
(EPS)

S3 Fig Validation of the common proximal interactors of the RAD51 paralogs. (A) Heatmap clustering representing the NormZ-scores of the common preys of the RAD51 paralogs in a series of CRISPR screens published by [23] where different genotoxic drugs were tested in RPE1-hTERT cells. Only cluster 1 indicated in **Fig 3B** is indicated. (B) GO biological processes (BP) analysis of the preys identified in cluster 1. Pathways are represented based on their p-value calculated by GO BP algorithm from cluster 1. Each dot is proportional to the number of preys that belong to the indicated pathway. (C) Schematic representing the CRISPR-based genome-wide screening pipeline developed to test the essentiality of a given gene in RPE1-hTERT cells. (D) Schematic representing the DR-GFP reporter assay. (E) Representation of the relative HR monitored in U2OS DR-GFP cells transfected with the indicated siRNA as published in [38].
(EPS)

S4 Fig Analysis of the BioID of the classical RAD51 paralogs completed under replication stress conditions. (A) Venn diagram representing the overlap of the different BioID of the RAD51 paralogs under HU and untreated conditions. (B) Heatmap clustering representing the NormZ-scores of the common preys of the RAD51 paralogs in a series of CRISPR screens published by [23] where drugs inducing replication stress were tested in RPE1-hTERT cells. Only cluster 1 indicated in Fig 4B is indicated. (C) GO biological processes (BP) analysis of the preys identified in cluster 1. Pathways are represented based on their p-value calculated by GO BP algorithm from cluster 1. Each dot is proportional to the number of preys that belong to the indicated pathway. (D) CladeOScope analysis of HIRA and CHERP in the Ascomycota phylum. (E) Representation of the percentage of S/G2 U2OS cells transfected with the indicated siRNA and displaying more than 9 IR-induced RAD51 foci as published in [44]. Each replicate is plotted on the x- and y-axis.
(EPS)

S5 Fig Several factors involved in RNA metabolic process that are proximal to the RAD51 paralogs have prognostic potential in BC. (A) Overall survival analysis based on the RNA levels of the indicated factors involved in RNA metabolic process in a cohort of BC patients using KMplot (n = 2976 patients). Only the preys linked to RNA metabolic process that have a significant impact on BC overall survival (p<0.05) are indicated.
(EPS)

S1 Table BioID of the classical RAD51 paralogs at steady state. (XLSX)

S2 Table Distribution of the overlap performed between the different BioIDs of the RAD51 paralogs. (XLSX)

S3 Table Characterization of the common proximal interactors of the BCDX2 complex (XLSX)

S4 Table Characterization of the common proximal interactors of the CX3 complex (XLSX)

S5 Table Characterization of the common proximal interactors of the RAD51 paralogs. (XLSX)

S6 Table Pathway enrichment analysis of the heatmap clusters obtained from the common proximal interactors of the classical RAD51 paralogs. (XLSX)

S7 Table Clustering of the different proximal interactors of the classical RAD51 paralogs alongside their respective CRISPR NormZ-scores. (XLSX)

S8 Table CRISPR essential screen performed in RPE1-hTERT cells. (XLSX)

S9 Table BioID of the classical RAD51 paralogs under HU treatment. (XLSX)

S10 Table Differential abundance of proximal interactors between HU and steady state conditions. (XLSX)

S11 Table Pathway enrichment analysis of the heatmap clusters obtained from the common proximal interactors of the classical RAD51 paralogs under HU treatment. (XLSX)

S12 Table Clustering of the different proximal interactors of the classical RAD51 paralogs under HU treatment alongside their respective CRISPR NormZ-scores. (XLSX)

S13 Table Characterization of the preys identified in cluster 1 from the BioID performed under HU treatment. (XLSX)

S14 Table RNA-seq (overall survival, OS; n = 2976) and gene chip analysis for relapse-free survival (RFS; n = 4934) and OS (n = 1880) of BC patients with a focus on preys linked to the spliceosome and RNA metabolic processes. (XLSX)
