# Supplementary material for: Systematic proximal mapping of the classical RAD51 paralogs unravel functionally and clinically relevant interactors for genome stability
Source: PLoS Genet. 2022 Nov 14;18(11):e1010495. doi: 10.1371/journal.pgen.1010495 (PMC9718398; doi:10.1371/journal.pgen.1010495)
Supplement: Attachment — Submitted filename: Point-by-point answers.pdf [file pgen.1010495.s020.pdf]

Point-by-point answers to the Reviewers' comments

Reviewer #1:

Cheyou et al present in this manuscript identification of proximal interactome of RAD51 paralogs using BioID technique under normal as well as replication stress conditions. RAD51 paralogs represent very interesting protein complexes involved not only in repair of double strand breaks, but also in processing of stalled replication forks. The latter being more likely associated also with genome instability and cancer development. In particular, RAD51C is also clinically relevant due to its association to breast cancer and Fanconi anaemia. However, very little mechanistic understanding of the role of RAD51 paralogs since their discovery over three decades ago have been provided, making this study very relevant. Nevertheless, the authors decided not to experimentally validate and provide mechanistic understanding of the identified linkages to various process, including spliceosome and RNA metabolic machinery. Instead, they utilised only publicly available datasets for their validation. This in my view represents the major hurdle in recommending this manuscript for publication in PLOS Biology.

We thank the Reviewer for his/her constructive comments. We are delighted that that Reviewer shares with us the need for an in-depth analysis of the different RAD51 paralogs and we agree that our study only provides a first hint at the cross-talks between these factors and other key molecular pathways. However, we believe that this study could represent a great resource to the field of genome stability.

Minor comments:

1. Tagging RAD51 and its paralogs have been associated with effects on their activities, therefore validation of the generated cell lines in respect of their sensitivity to DNA damage should be provided.

We thank the Reviewer for this comment. We shared with the Reviewer his/her concern about tagging the RAD51 paralogs; to validate our stable cell lines expressing BirA-Flag-tagged versions of the different RAD51 paralogs, we monitored the IC50 to cisplatin of our different cell lines using the sulforhodamine B (SRB) assay (Vichai et Kirtikara Nat Protocol 2006). As control, we targeted our parental cell line (HEK293T FLP-IN) with a siRNA against each respective paralog. These data are attached below and added in our revised manuscript as Figure S1C:

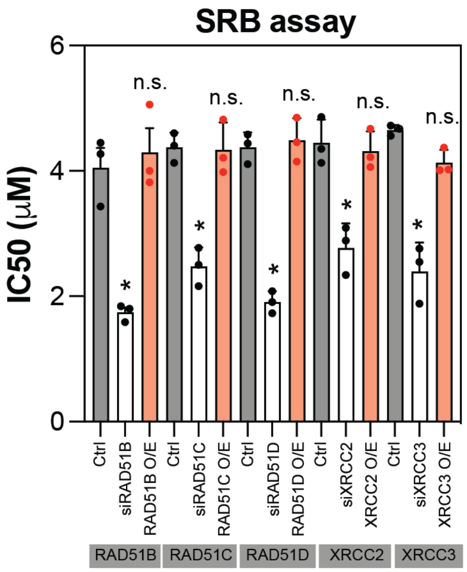

Figure 1. *Cell viability was monitored using the SRB assay in HEK293T FLP-In cells either transfected with a siRNA targeting the indicated RAD51 paralog or upon expression of the indicated paralog by tetracycline addition (n=3). Statistical analysis was performed using one way-anova test followed by Dunnett correction, * p>0.001.*

2. RAD51, well known interacting partner of RAD51 paralogs, has not been identified by this approach, any explanation why?

We thank the Reviewer for this great question. It remains unclear to us why we were not able to detect RAD51 in our different proximal interactomes. One possibility is that the N-terminal BirA tagging strategy that we employed to map the proximal interactome of the different RAD51 paralogs is beyond the ~10A radius to biotinylate RAD51.*

Reviewer #2:

In this manuscript Cheyou et al report proximal interactomes of the 5 classical RAD51 paralogs (RAD51B, RAD51C, RAD51D, XRCC2 and XRCC3) using the BioID approach in HEK293 cells. One interactome was obtained under "physiological conditions" and the other after exposure of cells to hydroxyurea to induce DNA replication stress.
In the first interactome analysis they find evidence for BCDX2, CX3 and RAD51C-PALB2-BRCA2 complexes which is consistent with previous studies. However, they uncover that RAD51C-PALB2-BRCA2 also interacts with RAD51D suggesting that other complexes of RAD51 paralog may form in cells. In addition, they identify PPIL2, C1orf112 as interesting partners that will be worth analyzing further for their roles in repair. Surprisingly, they identify spliceosome components in their interactome and they suggest that it could regulate RAD51 paralogs in their genome maintenance roles.
In the second screen, they identify many regulators of RNA metabolic processes. They postulate that these factors could be implicated in maintenance of genome integrity after DNA replication stress through functional interaction with the RAD51 paralogs.
The manuscript is well written and presented. The author put a lot of effort in intersecting the BioID information with data from various other screens and presenting the analysis in easy to ready graphics, which is not a trivial task. Overall, as potential new candidate regulators of the RAD51 paralogs are identified, the study will be of great interest to the field and will help further studies to unravel the roles of the RAD51 paralogs in genome maintenance mechanisms.

Major comment:
1. One concern about the study is that RAD51 paralog proteins are not very abundant in human cells. The way the authors have expressed the BirA fusions in HEK293 cells results in strong overexpression of the proteins. Could this overexpression have an impact on the interactome?

We thank the Reviewer for this comment. We employed the FPL-IN T-Rex system, which allows the stable integration of a single expression vection within the cell of interest. This system, while not ideal, still allowed the mapping of human cells (Go et al. Nature 2021) on the basis of 192 distinct subcellular markers. To ensure that our over-expression system did not alter significantly the interactome of the different RAD51 paralogs, we ensured that our proximal interactomes recapitulated known interactions, as detailed in Figure 1 of our manuscript.

It would have been preferrable to insert the BirA tag sequences in the endogenous gene locus in order to maintain physiological protein expression levels.

We agree with the Reviewer that the ideal approach would have been to endogenously tag our proteins of interest. When we initiated this project, this approach was not yet been optimized and we therefore decided to employ a system that has been robustly validated for the proximal mapping of several proteins.

Minor comments:

Figure 2H. For C1orf112, sensitivity for Olaparib is rather mild, isn't it?

C1orf112 has a normZ-score of -2.74 in SUM149PT cells and -2.41 in RPE1-hTERT, which is significant in our experience.

Figure 3G and S3E. The DR-GFP results for the RAD51 paralogs are surprising as they should be comparable to the BRCA2 phenotype except for RAD51B. Can we really trust these results?

We thank the Reviewer for this comment. We independently validated these data and we obtained a similar pattern in the U2OS DR-GFP cells. Therefore, we believe that we can trust this dataset:

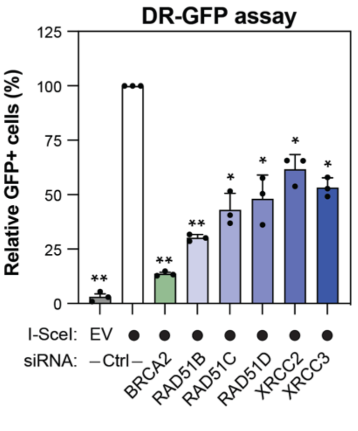

Figure 2. *U2OS DR-GFP were transfected with the indicated siRNA and either empty vector or a plasmid expression I-SceI (n=3). Statistical analysis was performed using one way-anova test followed by Dunnett correction, * p>0.05, ** p>0.001.*

Reviewer #3:
Cheyou et al have identified possible interactors of RAD51 paralogs using BioID-mass spectrometry and analyzed these putative interactors using CRISPR-mediated screening and previously published data sets. BRCA2 and PALB2 are confirmed as interactors, and the authors' analysis also provides evidence for RNA processing factors as an integral component of RAD51 paralogs' interactome. The authors suggest a linkage of RNA processing to DNA replication stress response and possible prognostic value of some of the identified interactors for breast cancer.

The main criticism is the lack of mechanistic studies to elevate the study beyond the descriptive level. For instance, is any of the RAD51 paralogs needed for recruitment of RNA processing factors to DNA damage or stressed DNA replication forks, or do the paralogs affect RNA processing per se? Absent mechanistic information, the information presented appears to be of archival value only.

Specific Comments:

1. It is unclear how the authors have addressed non-specific biotinylation in the Bio-ID experiments.

We thank the Reviewer for this comment. We removed classical BioID contaminants that are part of the CRAPome 2.0 repository, thereby excluding a total of 61 preys. We have clarified this step in the material and method section of our revised manuscript.

2. The authors have often used p-values to indicate significance of potential interactions with RAD51 paralogs for various proteins/gene groups from GO and KEGG analysis. However, it will be useful to compare absolute values such as SAF (spectral abundance factor) for interactor classes in box plots or comparable heatmaps presented as in Figs 3D and 4C, and comment on if higher scores suggest proximity.

We thank the Reviewer for this comment. We generated new tables that incorporated the SAF score for each of the interactor class (revised Supplementary Table 7 and revised Supplementary Table 12).

3. RAD51 paralogs have well-defined interactions among themselves and with other DNA repair factors (Fig. 1C). Therefore, these known interactors may be expected to show a higher score in the BioID screen. Accordingly, it will be useful to score interactions of newly identified partners against these already known interactors.

We thank the Reviewer for this comment. Our proximal mapping does not provide a score but rather an abundance of peptide, which can be reflective of many different variable, including the stability of the complex, the relative proximity to the bait and the detection of the peptide(s) by the MS device. Using peptide abundance as a sole criterion for scoring the relevance of a partner may not recapitulate its relevance for the complex. This is the reason why we incorporated chemogenomic profiling in our analysis as an additional way of stratifying our preys.

4. This study reports interactors for BCDX2 and CX3. However, it will be also useful to further examine shared vs novel partner proteins for BCDX2 and CX3. This would speak to distinct functions of the paralog complexes.

We thank the Reviewer for this comment. We have specifically performed this analysis in Supplementary Table 2 where we indicated the different preys per bait.

5. The association of RAD51 paralogs with the spliceosome and RNA metabolism machinery upon HU treatment may be indirect. Proper validation of physical interactions will be necessary to support major conclusions of the study.

We agree that our study only provides a first hint at the cross-talks between these factors and other key molecular pathways. However, we believe that this study could represent a great resource to the field of genome stability.

6. The authors found the E3 ubiquitin ligases PPIL2 and RAD18 among interactors of the RAD51 paralogs. Did they also detect ubiquitin in their BioID screen for any specific subunit of RAD51 paralogs?

We thank the Reviewer for this comment. While we agree with the Reviewer that it would very interesting to determine whether the different RAD51 paralogs get ubiquitylated, our current proteomic pipeline does not allow us to perform this analysis. Furthermore, we did not use any proteaosome and deubiquitinase inhibitor(s) while lysing our cells, therefore we may miss ubiquitylated products in our current analysis.
