# Supplementary material for: Systematic proximal mapping of the classical RAD51 paralogs unravel functionally and clinically relevant interactors for genome stability
Source: PLoS Genet. 2022 Nov 14;18(11):e1010495. doi: 10.1371/journal.pgen.1010495 (PMC9718398; doi:10.1371/journal.pgen.1010495)
Supplement: Attachment — Submitted filename: Revised_Cover_RAD51paralogs_Final.docx [file pgen.1010495.s021.docx]

Montreal, October 18^th^ 2022

**Vita Usova**

Publishing Editor, *PLOS Genetics*

Carlyle House, Carlyle Road

Cambridge CB4 3DN

United Kingdom

Dear Vita,

We just uploaded our revised manuscript entitled: **“Systematic proximal mapping of the classical RAD51 paralogs unravel functionally and clinically relevant interactors for genome stability”** on the online system of *PLOS Genetics* for your consideration. We made the following changes, in accordance with the suggestions of Reviewer #1:

1. We added the functional validation of our stable cells in Supplementary Figure S1 (c.f. Fig.S1C).
2. We updated the material and method section to describe the technique used in Fig.S1C, highlighted in yellow.
3. We updated the supplementary Figure legend to describe new panel, highlighted in yellow.

If there is any remaining matter that we have not addressed, please let us know and we will be happy to deal with it.

Best regards

**Dr. Alexandre Orthwein**, M.Sc., Ph.D.
Canada Research Chair in Genome Stability and Hematological Malignancies
Assistant Professor, Gerald Bronfman Department of Oncology, McGill University
Principal Investigator, Lady Davis Institute
3755 Chemin de la Côte-Sainte-Catherine, Montréal
QC, Canada H3T 1E2
Website: [orthweinlab.com](http://orthweinlab.com)
Email: [alexandre.orthwein@mcgill.ca](mailto:alexandre.orthwein@mcgill.ca)
Phone: (514) 340-8222 ext: 24252

**Dr. Barbara Rivera,** PhD,

Principal Investigator,

*Rare Tumors Lab - Hereditary Cancer Programme*

Adjunct professor, Gerald Bronfman Department of Oncology

Room BMC5

Bellvitge Biomedical Research Institute (IDIBELL)

Av Gran Via L’hospitalet, 199, 3rd Floor,

L’Hospitalet de Llobregat, Barcelona, Spain

Email: [brivera@idibell.cat](mailto:brivera@idibell.cat)

Phone: 936073800 – 3245
